# Increasing Proteome Coverage Through a Reduction in Analyte Complexity in Single-Cell Equivalent Samples

**DOI:** 10.1021/acs.jproteome.4c00062

**Published:** 2024-06-04

**Authors:** Marion Pang, Jeff J. Jones, Ting-Yu Wang, Baiyi Quan, Nicole J. Kubat, Yanping Qiu, Michael L. Roukes, Tsui-Fen Chou

**Affiliations:** † Division of Biology and Biological Engineering, California Institute of Technology, 1200 East California Boulevard, Pasadena, California 91125, United States; ‡ Division of Physics, Mathematics and Astronomy, California Institute of Technology, 1200 East California Boulevard, Pasadena, California 91125, United States; § Proteome Exploration Laboratory, Beckman Institute, California Institute of Technology, 1200 East California Boulevard, Pasadena, California 91125, United States; ∥ Division of Engineering and Applied Science, California Institute of Technology, 1200 East California Blvd, Pasadena, California 91125, United States

**Keywords:** single-cell proteomics, peptide identification optimization, protease choice, bottom-up proteomics

## Abstract

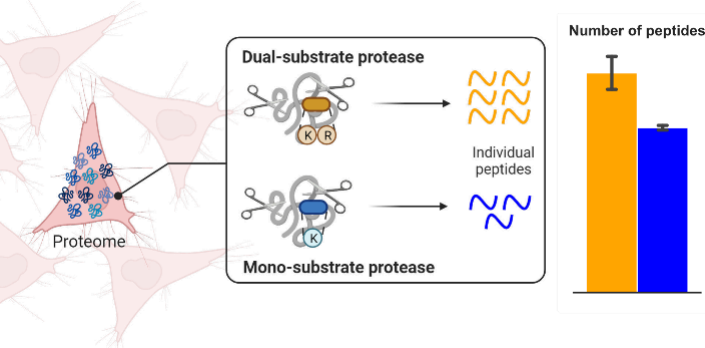

The advancement of sophisticated instrumentation in mass spectrometry has catalyzed an in-depth exploration of complex proteomes. This exploration necessitates a nuanced balance in experimental design, particularly between quantitative precision and the enumeration of analytes detected. In bottom-up proteomics, a key challenge is that oversampling of abundant proteins can adversely affect the identification of a diverse array of unique proteins. This issue is especially pronounced in samples with limited analytes, such as small tissue biopsies or single-cell samples. Methods such as depletion and fractionation are suboptimal to reduce oversampling in single cell samples, and other improvements on LC and mass spectrometry technologies and methods have been developed to address the trade-off between precision and enumeration. We demonstrate that by using a monosubstrate protease for proteomic analysis of single-cell equivalent digest samples, an improvement in quantitative accuracy can be achieved, while maintaining high proteome coverage established by trypsin. This improvement is particularly vital for the field of single-cell proteomics, where single-cell samples with limited number of protein copies, especially in the context of low-abundance proteins, can benefit from considering analyte complexity. Considerations about analyte complexity, alongside chromatographic complexity, integration with data acquisition methods, and other factors such as those involving enzyme kinetics, will be crucial in the design of future single-cell workflows.

## Introduction

Bottom-up proteomics, a mass spectrometry approach used for a majority of current proteomic studies, involves sequencing and identifying protease-derived peptides as proxies for full-length proteome constituents. Expanding the depth of coverage in single-cell proteomics poses a significant technical challenge, due to limited copy number per protein in single-cell samples, especially so for low abundance proteins. Notable progress has been made in single-cell proteomic sample preparation efforts. This includes, for example, the development of new methods and tools that enable the use of smaller sample volumes to minimize sample loss and increase reaction efficiency,^1−4^ improved throughput capabilities using new multiplexing methods such as that demonstrated in SCoPE-MS,^5^ and advances in tools for parallelizing sample preparation such as the ProteoCHIP^6^ and other techniques.^3,7,8^

However, a relatively unexplored facet in the context of single-cell proteomics are applications to reduce sample complexity, whereby we limit the total number of analytes both theoretically and in situ. Even within a single cell, protein copy numbers exhibit a considerable dynamic range with almost 6 orders of magnitude between the most abundant and least abundant proteins.^9−11^ The detection of abundant proteins often obscures the detection of biologically interesting low-abundance sequences, such as regulatory proteins,^12^ and the uncharted “dark proteome”.^13,14^ As a consequence, robustness, precision, and accuracy of the quantification process is diminished.^15−18^ Therefore, the development of strategies aimed at reducing oversampling of abundant proteins may improve the depth of quantitative precision and proteome coverage attainable in low analyte and single-cell proteomic samples.

Reducing complexity in proteomic analyses by way of wholly removing the most abundant proteins (i.e., depletion) is one method routinely applied to the investigation of complex mixtures, such as cellular lysates or plasma.^19^ Alternative strategies for mitigating analyte complexity include refining separation methodologies through advanced liquid chromatography and peptide fractionation techniques.^20−23^ Moreover, complexity reduction can be achieved through mass-spectrometry tools, such as parallel reaction monitoring (PRM) and ion mobility.^15,16,18^ It is important to note, however, that these approaches may either impose limitations on throughput or prove unsuitable for the analysis of sparse analyte samples and single-cell proteomes.^11,24,25^

**Figure 1:**
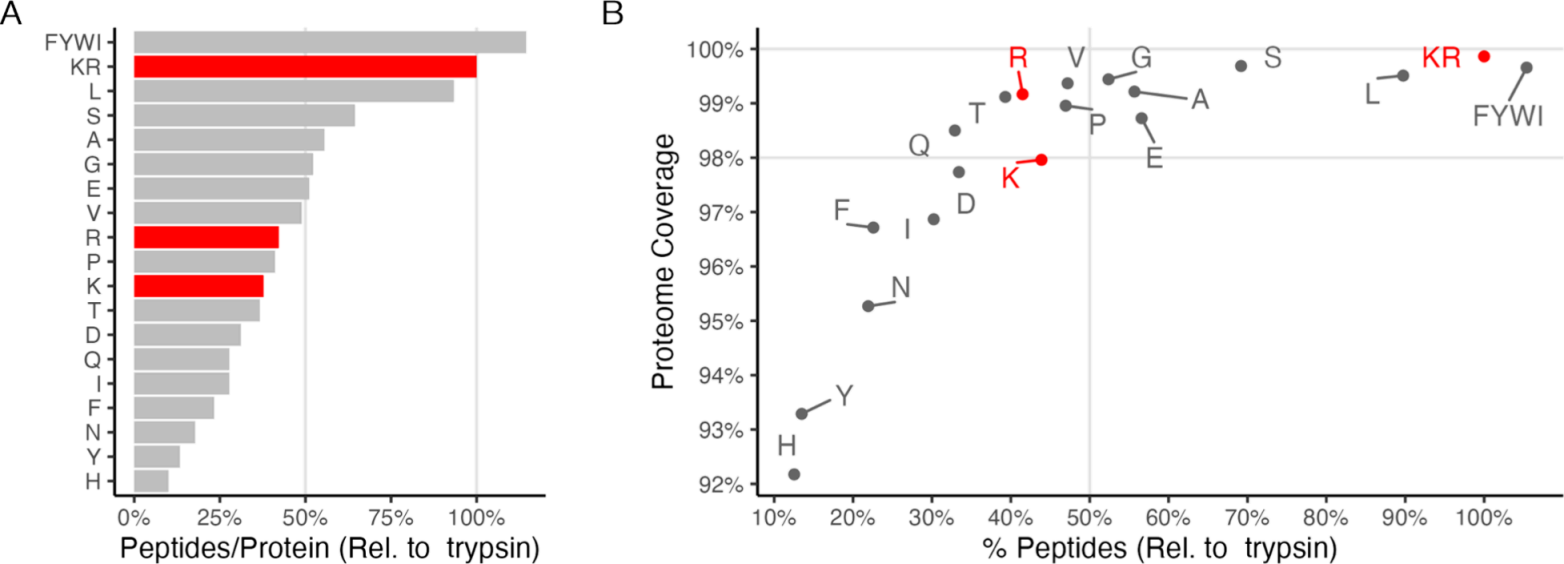
In silico simulation of protease digestion reveals that monosubstrate proteases maintain high proteome coverage while yielding fewer substrates (peptides per protein) compared to trypsin. A. Number of peptides (relative to trypsin) to total number of proteins yielding peptides within the criteria. Note, LysC (K) and ArgC (R) yield less than 50% the number of peptides compared to trypsin while still covering 98% of the human proteome or greater. B. The median number of peptides per protein, relative to trypsin, again demonstrating the potential for monosubstrate proteases to reduce protein oversampling compared to trypsin.

Trypsin is currently the most widely used protease in bottom-up proteomics, and such protocols have traditionally been optimized using bulk sample lysates.^26−28^ In the context of single-cell proteomics, various optimizations have been explored considering enzymes employed in the workflow including types of trypsin, substrate–enzyme ratios as well as combination of trypsin protease with detergents.^2,29,30^ Alternative proteases have recently been explored in the context of providing improved protein sequence coverage by purposefully oversampling individual protein sequences.^31,32^ However, these methods typically utilize large sample quantities and long duration chromotography to maximize analytical depth, these are not ideal for single-cell proteomics, where analytes are sparse and desirably high throughput. In high throughput proteomics, oversampling abundant proteins can diminish the total number of unique proteins identified in the sample, this is especially problematic in samples with limited analytes such as small tissue biopsies or single cells. To address this, we hypothesized that reducing the number of peptide analytes per protein basis would effectively reduce the analyte complexity of the sample, and thereby yield improved results for high-throughput single cell analyses. We explored, in silico, protease alternatives to trypsin, namely monosubstrate proteases, in an effort to identify proteases that generate fewer total peptides than trypsin for a given proteome, while maintaining similar proteome coverage. For the human proteome, LysC, which cleaves peptides C-terminal to lysine (K) residues, yields less than 40% as many peptides as trypsin (Figure 1A), which cleaves peptides C-terminal to both lysine and arginine (R), while still theoretically covering 98% of the known proteome (Figure 1B). Additionally, other proteases such as those cleaving only at glutamine (Q, GluC) or arginine also theoretically show similar low yields in total peptides while retaining an overall high proteome coverage. Trypsin, LysC, and GluC are all commercially readily available, and thus were chosen for further study to assess the potential experimental impact on proteome coverage.

In this study, we investigate the application of monosubstrate proteases, such as LysC, to mitigate the challenges associated with oversampling of abundant proteins in bottom-up proteomics assays.^33^ Our experimental findings demonstrate that using LysC results in identifying a similar number of proteins with significantly fewer peptides with improved quantitative accuracy, effectively reducing the overall sample complexity. Moreover, we explore the broader implications of this method in the context of single-cell proteomic methodologies, specifically examining the interplay of its effects on chromatographic complexity with varying LC run times, and analyte complexity, particularly considering the presence of carrier and reference proteomes. By elucidating the impact of monosubstrate proteases on analyte complexity, our study contributes to a comprehensive understanding of the interplay between experimental methodologies and sample complexity in single-cell proteomics. We conclude that monosubstrate proteases, such as LysC, can offer a significant advantage in terms of proteome coverage and quantitation accuracy for high throughput analysis of samples containing analyte amounts equivalent to single cells.

## Materials and Methods

### In Silico Simulation of Protease Digestion

A computational simulation was carried out to estimate the net reduction in complexity, when compared to trypsin, for a given protease (GluC: FYWI, trypsin: KR, LysC: K, ArgC: R) or n-terminal digesting for a specific amino acid using protein sequences from the human Uniprot database of proteins (20 398 sequences). The protease conditions were simulated to allow all possible 2 mis-cleaved events while only enumerating peptides with amino acid lengths between 6 and 60 residues, PTMs were not considered. All analyses were performed in R (R version 4.1.2, 2021–11–01) utilizing the package *msfastr*.^34^ Peptide and unique protein counts are shown Figure 1, demonstrating the differences in total number of peptides that account for a given proportion of the total proteome.

### Cell Culture

Human cell line HeLa S3 (CCL-2.2) was purchased from ATCC and grown as adherent cultures in 10 cm plates and maintained in DMEM (Sigma-Aldrich) supplemented with 10% (v/v) fetal bovine serum, glutamine (2 mmol/L), penicillin (100 IU/ml), and streptomycin (100 IU/ml). Passage of HeLa cells was conducted every 2 days, typically when cell density reached approximately 10^6^ cells/ml. TrypLE Express (Thermo Fisher Scientific) was used for cell harvesting with gentle pipetting. Cell suspensions were then washed with cold phosphate-buffered saline and subjected to centrifugation at 300*g* for 4 min, and the supernatant discarded. Pellets, containing approximately 2 × 10^6^ cells each, were then promptly frozen at −80 °C until further use, prior to cell lysis and subsequent digestion.

### Sample Preparation for Mass Spectrometry

Identical sets of samples were subjected to two independent workflows; one that diluted samples prior to protease digestion (dilute-then-digest) and another that subjected samples to an optimal protease digestion, with respect to sample-protease ratio and reaction volumes, prior to dilution (typical bulk digestion).

Lysis buffer (500 μL) consisting of 50 mM triethylammonium bicarbonate (TEAB) (Thermo Scientific, 90114) and 0.1% *n*-Dodecyl-β-Maltoside (DDM) (Thermo Scientific, 89903) was added to each cell pellet. Each pellet was then gently pipetted, followed by sonication using a Branson 550 probe sonicator for five rounds of 3 s, 10 J, pulses at 60% amplitude to achieve cell lysis. Samples were then heated for 1 h at 70 °C with the thermocycler’s heated lid set to 105 °C for protein denaturation. Finally, samples were centrifuged at 3000 rpm, and the protein concentration in the lysate was determined using a Pierce BCA Protein Assay kit (Thermo Scientific, 23225).

For serial dilution of bulk digested samples, samples were diluted with freshly prepared Solvent A (comprising 97.8% water, 2% acetonitrile, 0.2% formic acid (FA)) with 0.1% DDM. For samples prepared using the dilute-then-digest method, serial dilutions were prepared at protein concentrations of 100 ng/μL, 20 ng/μL, 2 ng/μL, and 200 pg/μL using Solvent A with 0.1% DDM. 100 μL of each dilution was then aliquoted into wells of a nonskirted 96-well PCR plate (Thermo Scientific, AB0600). The remaining 100 ng/μL sample was used for the preparation of digest-then-dilute samples and aliquoted 300 μL in Protein LoBind 1.5 mL tubes (Eppendorf, 022431081).

Proteolytic digestions were carried out using Glu-C (Thermo Fisher Scientific), Lys-C (Wako Chemicals, Lysyl Endopeptidase), and trypsin (Thermo Fisher). For samples prepared using the dilute-then-digest method, 2 μL of enzyme was added to each sample, resulting in a final 1:10 enzyme–substrate ratio per protein concentration. For samples prepared using the digest-then-dilute methods, 6 μL of each enzyme (500 ng/μL) was added to each aliquot for a 1:10 enzyme to substrate ratio. Both sets of samples were then incubated at 37 °C overnight. Following digestion, samples were centrifuged at 1000*g* for 1 min, and digestion was quenched with 1 μL of Solvent A with 4% FA. Peptide concentration was determined using a Pierce Quantitative Fluorometric Peptide Assay kit (Thermo Scientific, 23290), and serial dilutions using Solvent A with 0.1% DDM were performed for digest-then-dilute samples to generate samples with peptide concentrations of 100 ng/μL, 20 ng/μL, 2 ng/μL, and 200 pg/μL, which were subsequently added to a 96-well plate.

### LC-MS/MS Analysis

Peptides were separated on an Aurora Ultimate UHPLC Column (25 cm by 75 μm, 1.7 μm C18; AUR3–25075C18, IonOpticks) with column temperature maintained at 50 °C. To optimize system sensitivity, peptides were directly introduced onto the analytical column without the use of a trapping column. The separation gradient was configured with a flow rate of 0.22 μL/min for all gradients. For digestion methods and dilution series, samples were run using a gradient run time of 50 min (including washing) unless otherwise noted. The LC system (Vanquish Neo UHPLC, Thermo Scientific) was coupled to an Orbitrap Exploris 480 mass spectrometer (Thermo Scientific) with a Nanospray Flex ion source (Thermo Scientific). Data-dependent acquisition (DDA) was carried out in positive ion mode using a positive ion voltage of 1600 V while maintaining the ion transfer tube at a temperature of 300 °C. MS1 scans were acquired with a range of 375–1200 *m*/*z* and a resolution of 60 000 with a cycle time of 3 s. The maximum injection time was set to auto, and the normalized AGC target was set to 300%. Precursor ions with charges ranging from +2 to +6 were selectively targeted for fragmentation using a minimum intensity threshold of 5e3. Dynamic exclusion was set to exclude after one acquisition, with a 45 s exclusion duration and 10 ppm mass tolerance. MS2 scans were acquired in the Orbitrap at 60 000 resolution with a isolation window of 1.6 *m*/*z*, HCD collision energy set at 28%, and an autoadjusted maximum injection time. The normalized AGC target was set at 200%. Xcalibur software (Thermo Scientific) was used for method implementation and data acquisition. Further information on the gradient conditions and MS settings can be found in the Supporting Information (SI Tables S1 and S2).

### Mass Spectrometry Data Analysis and Statistics

Proteome Discoverer 2.5 (Thermo Fisher Scientific) was used to analyze RAW files using the SequestHT^35^ search algorithm with Percolator validation. For the individual samples, each of the three respective enzymes was selected, allowing for a maximum of two missed cleavages and peptide lengths between seven and 30 amino acids. The mass tolerance for precursor ions was set at 20 ppm, while the fragment mass tolerance was defined as 0.1 Da. Carbamidomethylation of cysteine residues was designated as a static modification, and oxidation of methionine residues was considered a dynamic modification. INFERYS Rescoring was used in automatic mode, and Percolator was used for search validation based on *q*-values with a strict false discovery rate (FDR) of 1% at the spectrum level.^36,37^ A minimum number of 1 peptide sequence (unique and razor) was required for protein identification. Proteins meeting a stringent FDR threshold of 0.01 were assigned as “high confidence” and used for further analysis. Strict parsimony was used to group proteins. Data was exported from PD2.5 and used for further analysis. The RAW data have been deposited to the ProteomeXchange Consortium via the PRIDE^38^ partner repository with the data set identifier PXD048926.

**Figure 2:**
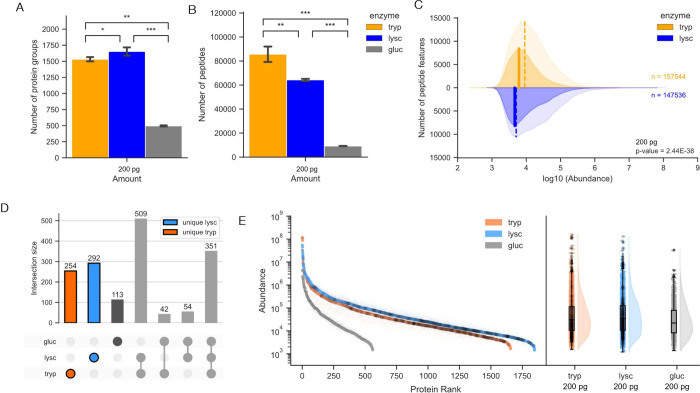
Comparison of monosubstrate enzymes versus trypsin used to digest a 200 pg HeLa lysate (*n* = 3). Two μL of each sample was used for LC/MS-MS analysis as described in the methods, with 200 pg representative of a single cell equivalent load; see also SI Figures S1 and S2). A. Average number of protein groups identified with high confidence. (q<0.01) B. Number of peptides identified. C. Consensus feature counts plotted for trypsin (orange) and LysC (blue) that were either extracted only (light shading) or further identified (dark shading). Dotted and solid lines denote medians for extracted and identified features, respectively. D. UpSet plot indicating intersection sizes between proteins identified for samples digested with trypsin, LysC and GluC. E. Quantitative rank plot of proteins identified. Proteins uniquely identified in samples prepared using trypsin or LysC are outlined in black in D, and colored black in E.

**Figure 3:**
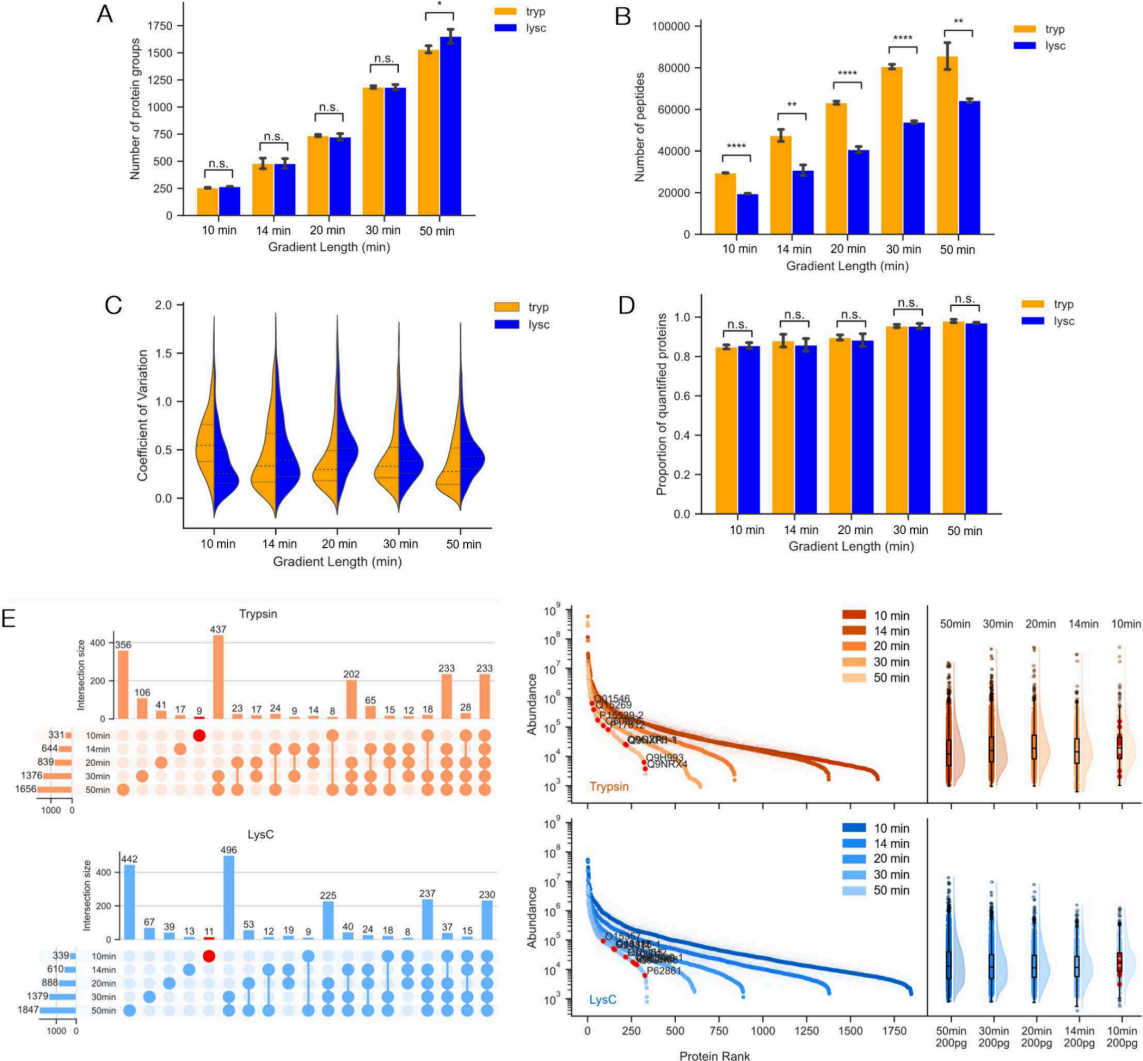
Bottom-up proteomics analysis of HeLa cell lysates (200 pg sample load) digested with three different enzymes (trypsin, LysC, GluC) analyzed with five different LC gradient run times (10, 14, 20, 30, 50 min (*n* = 3); see also SI Table S1, Figures S4–S6). A. Number of quantified proteins (q<0.01) and B. peptides (q<0.01) across different LC gradient run times. C. CV distributions and D. proportion of quantified peptides. E. Quantitative rank plots for the same sample runs. Proteins identified exclusively using the shortest (10 min) gradient time are denoted in red.

**Figure 4:**
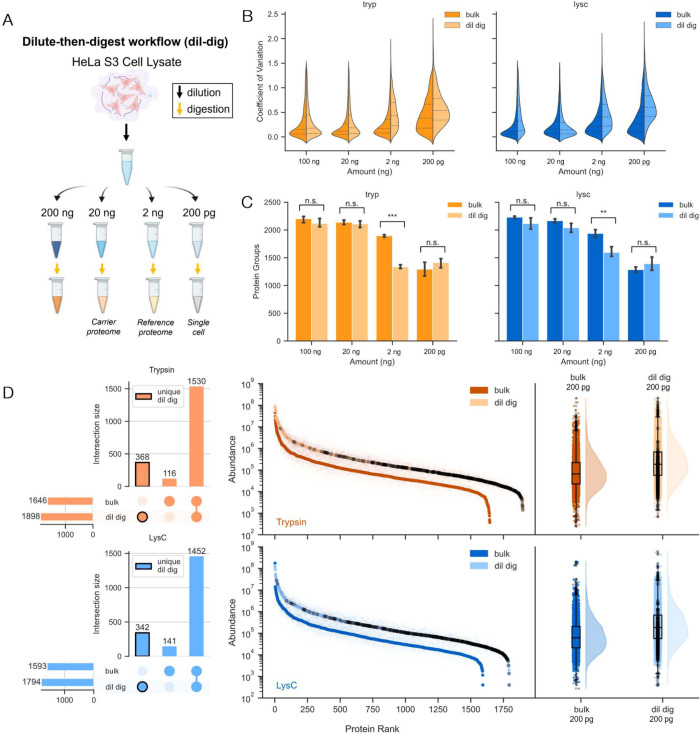
Effect of using different input analyte amounts for digestion reactions performed using either LysC or trypsin (*n* = 8); see also SI Figure S7–S9). A. Bottom-up proteomics analysis of low analyte samples was performed on HeLa S3 lysates using two distinct procedures: digestion of bulk lysate prior to serial dilution (bulk digests), or serial dilution of bulk lysate prior to digestion (dilute-then-digest, or dil-dig as illustrated). Bulk digest 20 ng and 2 ng samples are reflective of the preparation methods similar to that for carrier and reference proteomes, respectively. B. Plot of CV distributions for both enzymes, comparing the dilute-then-digest (lighter color) and bulk digest (darker color) methods. C. Number of proteins identified with high confidence for the same samples. (q<0.01) D. Quantitative rank plots of proteins identified in the 200 pg (single cell) load for the two methods. Proteins identified exclusively in a single method are noted in black.

Proteomics data was exported into the R (R version 4.1.2, 2021–11–01) tidyproteomics^39^ package for normalization and tidying. LCMS features were extracted and analyzed using the Dinosaur^40^ software package. Further data analysis and plots were done in Python. The UpsetPlot^41^ package was used to generate upset plots in Figures 2, 3, and 4. Identified and extracted consensus features were exported from consensus information in PD2.5. Kolmogorov–Smirnov (KS) normality test was used to test distribution for whether proteins unique to certain conditions lay within the original sample distribution.

## Results and Discussion

### Reduction in Oversampling of Peptides Facilitated by Monosubstrate Protease at Single-Cell Equivalent Levels

To explore possible advantages of using a monosubstrate protease for bottom-up proteomics analysis of single cell samples, we compared results obtained in bottom-up proteomics studies of HeLa cell lysates performed using either trypsin or a monosubstrate protease (LysC, GluC) and single-cell equivalent protein loads (200 pg). In terms of number of proteins identified, LysC significantly outperformed optimized trypsin by almost 10% (7.8%, student’s *t* test, *p* < 0.05; Figure 2A). Furthermore, in agreement with in silico analyses (Figure 1B), fewer peptides were identified in samples prepared using the monosubstrate proteases tested compared to trypsin (LysC generating 75% the number of peptides generated by trypsin, Figure 2B). Similarly, the median abundance of peptide features extracted from mass spectrum and subsequently matched to a peptide sequence for monosubstrate proteases was also statistically less compared to trypsin (Figure 2C; Wilcoxon rank-sum test, *p* < 0.05, LysC; GluC data not shown). Taken together, these results support the hypothesis that reducing analyte complexity can translate to maintaining or slightly improving depth-of-coverage per feature/analyte extracted in bottom-up proteomics studies by reducing analyte complexity through reducing oversampling of peptides.

We note that unlike LysC, samples prepared using GluC had significantly fewer protein groups identified than samples prepared with either LysC or trypsin, potentially due to GluC cleavage leaving acidic residues, which suffer from ionization.^42^ This drop in performance was observed not only at the protein group level but also in the number of detected peptides (Figure 2A, B). We acknowledge that beyond considering the monosubstrate nature of a protease, there is a need to carefully evaluate intrinsic properties of different enzymes, to determine their suitability across diverse mass spectrometry applications. These properties may yield varied outcomes, as discussed in previous studies,^28,42^ reflecting the nuanced role of protease choice beyond its impact on analyte complexity in mass spectrometry applications.

In terms of proteins identified, there were 254 and 292 proteins uniquely identified in samples prepared using LysC and trypsin, respectively (Figure 2D). Notably, these proteins appear evenly spread across protein ranks regardless of protease (Figure 2E) (KS test p-val of 7.2 × 10^–8^, 6.1 × 10^–7^ for trypsin and LysC, respectively), suggesting that the identification of unique proteins was due to stochastic sampling facilitated by mass spectrometry data-dependent acquisition (DDA). Thus, the choice of protease does not appear to manifest bias toward proteins of either high or low abundance. Additionally, this observation signifies that the current sensitivity of mass spectrometry is indeed adequate for single-cell proteomics, emphasizing that stochastic sampling through DDA may act as a limiting factor in pushing the boundaries of single-cell protein identification. This lends strength to the argument for a data-independent acquisition (DIA)^43^ approach to single-cell proteomics, where all precursors are fragmented and analyzed, and random sampling is no longer a factor, which addresses the limitations of DDA in terms of inherent irreproducibility and under-sampling. Preliminary experiments conducted on single-cell equivalents using DIA corroborate this assertion (Figure S3). Furthermore, recent studies have explored the utility of DIA in single-cell proteomics, and its potential in terms of addressing fundamental questions in cell biology.^8,44−46^

### Analyte Complexity in the Context of Chromatography Duration

When delving into single-cell proteomic techniques, which often aim to characterize heterogeneity within a population, it becomes crucial to increase throughput by either simultaneously analyzing more samples, or by decreasing LCMS run time spent on each sample. The former can be achieved using techniques involving multiplexing, such as employing isobaric tags such as tandem mass tags (TMT)^47^ and isobaric tags for relative and absolute quantification (iTRAQ).^48^ Conversely, the latter, though it ensures more runs per unit time, also inherently increases analyte complexity in a given elution window, since reducing LC separation time results in peptides with closer retention times, and thus more peptides per elution window, placing a heightened demand on the MS for efficient peptide separation and identification.

In the context of single-cell proteomics, various studies have explored the optimization of minimal gradient times,^20,49,50^ showing that analyte complexity typically increases with a decreased gradient time. Motivated by these findings, we sought to assess whether the reduction in peptides generated by monosubstrate proteases could offset the increase in complexity of analytes entering the MS at a given time. We hypothesized that the combination of monosubstrate proteases with decreased gradient run times may yield superior outcomes compared to trypsin, particularly for studies focusing on sparse analytes, such as those encountered in single-cell bottom-up proteomics.

To test this, we performed proteome profiling of single-cell equivalent samples (200 pg) using varying LC gradient times (10, 14, 20, 30, and 50 min). As expected, the longest (50 min) LC gradient time yielded the greatest number of identified proteins groups and peptides for both enzymes used for sample preparation (Figure 3A, B). Further, LysC showed a statistically significant advantage in quantitative accuracy with a smaller coefficient of variation (CV) at shorter gradient lengths compared to trypsin (Figure 3C), with similar performance to trypsin in terms of the proportion of quantified proteins across LC gradient times tested (Figure 3D). The improvement in CVs is noted in both unique and shared peptides at the 10 min gradient (Figure S5). Closer inspection of proteins identified exclusively in 10 min LC runs revealed fairly uniform distributions across the abundance-rank plot (Figure 3E). Further, despite the constraints imposed by a short acquisition time, where chromatographic separation is minimized, the mass spectrometer nonetheless identified proteins across a broad dynamic range.

Taken together, we demonstrate a consistent reduction in analyte complexity achieved through the use of the monosubstrate protease LysC across decreasing gradient lengths. However, this reduction does not necessarily translate to improved performance, given the multifaceted nature of MS-based proteomics, which involves factors including chromatographic complexity, notably LC time, among others. It is noteworthy that our approach offers advantages in terms of statistical confidence, as evidenced by comparable or superior coefficient of variations (CVs) while maintaining quantitation.

### Analyte Complexity in the Context of Carrier and Reference Proteomes Used for Single-Cell Methodologies

Carrier/reference proteomes are integral components of single-cell proteomic experiments, strategically employed to enhance sensitivity and depth of identification. Initially introduced in Budnik et al.,^5^ the use of booster or carrier/reference channels has become a prevalent strategy employed in conventional multiplexed single-cell experiments in literature.^51−55^ These channels typically involve protein amounts equivalent to 100–200 cells and 5–20 cells, respectively, which are subsequently subjected to digestion. The strategy seeks to improve single-cell mass spectrometry by minimizing sample loss and enhancing peptide identifications, thus improving the depth of identification for single cell samples. In addition to their utility, carrier/reference proteomes also introduce additional layers of analyte complexity. High carrier proteome concentrations that cause ion coalescence and space charging have been identified as potential factors that can impact quantitative accuracy of single-cell proteomics data using such an approach, an observation that is commonly attributed to dynamic range and the carrier proteome effect on sampling of single-cell ions.^56−60^ This phenomenon underscores the intricate interplay between analyte complexity and experimental methodologies, highlighting the need for a comprehensive understanding of these factors in single-cell proteomics research. Moreover, variables such as enzyme reaction conditions, reaction volume, and surface exposures play crucial roles in single-cell proteomic workflows, and their potential differences between proteolytic reactions using carrier/reference samples and those at the single-cell level should be considered. In particular, the digestion step of such sample preparation approaches could be an additional factor that potentially affects quantitative proteomics.^61−64^ Furthermore, any differences in these variables that lead to systematic sample losses during processing could also introduce biases in observed results.

In light of these considerations, one aim of our study is to investigate the impact of input protein quantity during the digestion process on analyte complexity, with the aim of understanding their effect on multiplexing strategies in single-cell proteomic workflows. We hypothesized that input protein quantity during the digestion process may impact analyte complexity, and result in different peptide populations. Moreover, we investigate the potential benefits of protease selection in conjunction with variations in input protein quantities during the digestion process, particularly focusing on their role in reducing analyte complexity through the utilization of monosubstrate proteases. To test this hypothesis, we introduced a sample preparation methodology designed to more accurately replicate the conditions encountered in the preparation of single cells, reference channels, and carrier channels for bottom-up proteomics experiments. Our approach involved a comparative analysis between a diluted bulk digest and cellular lysates digested at varying input amounts, employing a dilute-then-digest strategy (Figure 4A), in which HeLa cellular lysate was diluted to various input amounts for digestion — 200 pg (single cell equivalent sample), 2 ng (10-cell equivalent sample, typical of a reference channel in multiplexed single-cell experiments), 20 ng (100-cell equivalent sample, typical of a carrier channel in multiplexed single-cell experiments), and 200 ng (bulk load). We also explored the use of both disubstrate and monosubstrate enzymes to assess potential advantages in terms of analyte complexity.

Low CVs were observed across the dilution series in bulk-digested samples, regardless of protease used, reflective of small differences in experimental handling accuracy when performing dilution (Figure 4B). Interestingly, dilute-then-digest samples showed larger variability at lower analyte amounts (2 ng and 200 pg), suggesting that additional factors affecting digestion kinetics or other digestion reaction related factors may additionally contribute to the increased variability observed, especially at single cell concentrations. Samples diluted from a bulk digest, on the other hand, have a similar proteome coverage to dilute-then-digest samples at high concentrations, except at low analyte 2 ng loads with a 20% increase in identified proteins, as illustrated in Figure 4C. Furthermore, we note that LysC significantly outperforms trypsin at the bulk single cell equivalent 200 pg load in lower CVs for both shared overlapping peptides (KS test *p*-val 2.8 × 10^–4^), as well as unique peptides (KS test *p*-val 1.3 × 10^–33^) (Figure S7).

We note that the number of protein groups and peptides identified plateaued at a 20 ng load, with no significant improvement at the 100 ng load (Figure 4C, SI Figure S6A). This observation could be attributed to the relatively short 50 min gradient time and MS/MS AGC set at 200%, which was optimized for single-cell sensitivity and higher throughput. Similar observations of protein identification plateaus at high loads have also been reported in previous studies.^49,50^ We also note that CV of the proteins identified increase with decreasing loads, with the lowest CV observed at the 100 ng and 20 ng loads and an increase observed at lower protein loads (Figure 4B). This trend aligns with the expectation that higher input amounts improve ion statistics, thereby contributing to more confident quantification compared to lower input samples.

Further exploring the 200 pg loads, we compare the two different sample preparation methods across the two different enzymes. Specifically, proteins that were uniquely identified in the dilute-then-digest samples were fairly uniformly distributed across protein ranks, indicating that any sample loss, or differences arising from sample preparation, can affect a large part of a proteome’s dynamic range. Proteins exclusively identified in samples prepared using the dilute-then-digest approach also appeared to be slightly biased toward the lower abundance side of dynamic range (KS test *p*-val of 1.8 × 10^–15^, 2.3 × 10^–15^ for trypsin and LysC, respectively).

Moreover, we acknowledge that enzyme kinetics is affected by a multitude of factors in addition to sample dilution. Factors such as the origin of the protease source, enzyme–substrate ratios, molecular enzyme concentration within the volume, and synergistic combinatorial effects of multiple proteases contribute significantly to enzymatic efficiency.^7,29,65^ Noteworthy studies by Wang et al.^7^ and Woessmann et al.^65^ have demonstrated that enzyme–substrate ratio has a significant impact on both quantitative and qualitative performance, with enhanced peptide yields. These investigations proposed hypotheses regarding the efficacy of high enzyme concentrations within small volumes to increase digestion efficiency or to serve as adsorption substitutes, thereby reducing adsorption loss. Although the present study investigates the input amount during digestion while controlling for these factors, further research is warranted to comprehensively characterize the interplay among these multivariate factors. Future endeavors in single-cell sample preparation workflows, particularly those involving multiplexing experiments with carrier proteomes, should carefully consider these factors when designing experiments.

## Conclusions

Collectively, the results of our study collectively indicate that for samples with fewer analytes, such as small tissue biopsies or single-cell specimens, the use of a monosubstrate protease such as LysC and potentially ArgC, can offer advantages in terms of proteome coverage and enhanced quantitative precision for high-throughput proteomic analyses. However, apart from considering the monosubstrate nature of a protease, its intrinsic properties must be carefully evaluated to assess suitability across diverse mass spectrometry applications. These properties, as discussed in previous studies,^28,42^ may yield varied outcomes, thus highlighting the nuanced role of protease choice beyond its impact on analyte complexity in mass spectrometry applications. The analytical challenges associated with oversampling are a persistent and complex hurdle in mass spectrometry, hence developing simple and effective methods to address this challenge is crucial.

Our findings reveal the potential advantages of using a monosubstrate protease for high-throughput bottom-up proteomics experiments with limited analyte quantity. By generating fewer MS analytes (peptides), it is possible to achieve improved quantitative accuracy at relatively short chromatography gradient times while maintaining or slightly improving depth-of-coverage per feature/analyte. Notably, we observed an enhanced coefficient of variation (CV) when comparing LysC to trypsin at single-cell equivalent loads for both shared and unique peptides. Taken together, this suggests that using LysC, or possibly another monosubstrate protease such as ArgC, may be advantageous for high-throughput single-cell applications. Furthermore, regarding the impact of using carrier proteomes, commonly associated with dynamic range effects and the detection of single-cell ions, our study introduces an additional factor for consideration: the input amount during digestion, which may affect downstream MS analyte complexity by nature of the types of peptide generated. This may be an important consideration for the design of carrier proteomes in future multiplexing experiments. It suggests that the analyte complexity of carrier or reference proteomes could be influenced by digestion conditions. Moreover, enzyme kinetics are influenced by various factors beyond sample dilution and input amounts, including protease origin, enzyme–substrate ratios, and molecular enzyme concentration. While our study addresses input amount during digestion while controlling for these factors, further research is needed to fully understand their interplay among these multivariate factors. Future single-cell workflows should consider these factors in experiment design.

The outcomes of this investigation were derived through shotgun data-dependent acquisition. This methodology is intrinsically skewed toward high-abundance precursors, and is further challenged by stochastic sampling and the potential for unidentifiable precursors. To address these challenges, analyte complexity strategies can be used in tandem with innovative data acquisition methods such as pSCoPE,^66^ which employs prioritized analysis to mitigate the aforementioned challenges, thereby augmenting both data comprehensiveness and proteome coverage depth. Future research avenues may involve exploring protease selection in tandem with advanced acquisition techniques such as data-independent acquisition (DIA),^43,45^ which help to mitigate the limitations of DDA such as irreproducibility and under-sampling. Recent studies have explored the efficacy of DIA in single-cell proteomics, demonstrating its potential to address fundamental inquiries in cell biology and yielding promising results, with some investigations also integrating multiplexing strategies with DIA to enhance its utility and versatility in proteomic analyses.^8,44,46,49,67^

Future research could further explore and take advantage of these findings in practical applications, particularly those using isolated single cells. This work also highlights the need to explore alternative proteases for bottom-up proteomics methods. Our in silico findings suggest that monospecific proteases specific to arginine (R) and glutamine (Q) may be good candidates for additional studies. Investigating these proteases may further help to optimize bottom-up proteomic analyses used for analyzing samples with low analyte densities. They could facilitate future breakthroughs in our understanding of cellular processes at the single-cell level, and thereby provide deeper insights into cellular heterogeneity and the molecular underpinnings of complex biological phenomena.

## Data Availability

PD exports, mass spectrometry RAW data and lists of unique proteins identified in Figure 2E, Figure 3E and Figure 4D have been deposited to the ProteomeXchange Consortium via the PRIDE partner repository with the data set identifier PXD048926.

## Supporting Information Available

Table S1, Gradient conditions for LC-MS/MS analysis; Table S2, MS settings for DIA LC-MS/MS analysis; Figure S1, further characterization of proteomic data from different proteases; Figure S2, LCMS experimental details from different proteases; Figure S3, proteomic DIA data from different proteases at single-cell equivalent; Figure S4, further characterization extraction and identification of peptide features across various acquisition times; Figure S5, LCMS experimental details from different proteases (LysC, trypsin) across various acquisition times; Figure S6, CV distributions of shared and unique peptides across various acquisition times; Figure S7, further characterization of proteomics data from bulk digest and dil-dig Tryp and LysC; Figure S8, CV distribution for peptides that shared and unique peptides across bulk digest and dilute-then-digest methods at single-cell equivalent; and Figure S9, LCMS experimental details from bulk digest and dilute-then-digest methods across various input loads at digestion (PDF)
