# Supplementary material for: Increasing Proteome Coverage Through a Reduction in Analyte Complexity in Single-Cell Equivalent Samples
Source: J Proteome Res. 2024 Jun 4;24(4):1528–38. doi: 10.1021/acs.jproteome.4c00062 (PMC11976869; doi:10.1021/acs.jproteome.4c00062)
Supplement: Supplementary file 1 — pr4c00062_si_001.pdf [file pr4c00062_si_001.pdf]

Supporting Information

Increasing Proteome Coverage Through a Reduction in Analyte Complexity in Single-Cell Equivalent Samples

Marion Pang[1,*], Jeff J Jones[2], Ting-Yu Wang[1,3], Baiyi Quan[1,3], Nicole J Kubat[2], Yanping Qiu[1,3], Michael L Roukes[1,2,*], Tsui-Fen Chou[1,3,*]

[1]Division of Biology and Biological Engineering, California Institute of Technology, 1200 E. California Blvd., Pasadena, CA 91125

[2]Division of Physics, Mathematics and Astronomy, California Institute of Technology, 1200 E. California Blvd., Pasadena, CA 91125

[3]Proteome Exploration Laboratory, Beckman Institute, California Institute of Technology, 1200 E. California Blvd., Pasadena, CA 91125

*Correspondence to: marion.pang@caltech.edu, roukes@caltech.edu, tfchou@caltech.edu

Table of Contents

Supplemental Table S1. Gradient conditions for LC-MS/MS analysis S2

Supplemental Table S2. MS settings for DIA LC-MS/MS analysis S3

Supplemental Figure S1. Further characterization of proteomic data from different proteases S4

Supplemental Figure S2. LCMS experimental details from different proteases S4

Supplemental Figure S3. Proteomic DIA data from different proteases at single-cell equivalent S4

Supplemental Figure S4. Further characterization extraction and identification of peptide features across various acquisition times S5

Supplemental Figure S5. LCMS experimental details from different proteases (LysC, trypsin) across various acquisition times S6

Supplemental Figure S6. CV distributions of shared and unique peptides across various acquisition times S7

Supplemental Figure S7. Further characterization of proteomics data from bulk digest and dil-dig Tryp and LysC S7

Supplemental Figure S8. CV distribution for peptides that shared and unique peptides across bulk digest and dilute-then-digest methods at single-cell equivalent S8

Supplemental Figure S9. LCMS experimental details from bulk digest and dilute-then-digest methods across various input loads at digestion S9

Supplemental Table S1. Gradient conditions for LC-MS/MS analysis

50 min gradient

Time (min)	Duration (min)	Flow rate (μl/min)	% Solvent A	% Solvent B
**0.0**	0.0	0.220	97.0	3.0
**1.0**	1.0	0.220	97.0	3.0
**31.0**	30.0	0.220	81.0	19.0
**41.0**	10.0	0.220	71.0	29.0
**51.0**	10.0	0.220	59.0	41.0
**52.0**	1.0	0.220	5.00	95.0
**52.0**	**Column Wash**			
**57.0**	5.0	0.220	5.0	95.0
**57.0**	**Stop Run**			
**57.0**	**Column Equilibration**			

30 min gradient

Time (min)	Duration (min)	Flow rate (μl/min)	% Solvent A	% Solvent B
**0.0**	0.0	0.220	99.0	1.0
**0.5**	0.5	0.220	99.0	1.0
**1.0**	0.5	0.220	92.0	8.0
**24.0**	15.0	0.220	80.0	20.0
**30.0**	4.0	0.220	65.0	35.0
**30.0**	**Column Wash**			
**34.0**	4.0	0.220	1.0	99.0
**34.0**	**Stop Run**			
**34.0**	**Column Equilibration**			

20 min gradient

Time (min)	Duration (min)	Flow rate (μl/min)	% Solvent A	% Solvent B
**0.0**	0.0	0.220	99.0	1.0
**0.5**	0.5	0.220	99.0	1.0
**1.0**	0.5	0.220	92.0	8.0
**16.0**	15.0	0.220	80.0	20.0
**20.0**	4.0	0.220	65.0	35.0
**20.0**	**Column Wash**			
**24.0**	4.0	0.220	1.0	99.0
**24.0**	**Stop Run**			
**24.0**	**Column Equilibration**			

14 min gradient

Time (min)	Duration (min)	Flow rate (μl/min)	% Solvent A	% Solvent B
**0.0**	0.0	0.220	99.0	1.0
**0.5**	0.5	0.220	99.0	1.0
**1.0**	0.5	0.220	92.0	8.0
**11.2**	1.02	0.220	80.0	20.0
**14.0**	2.8	0.220	65.0	35.0
**14.0**	**Column Wash**			
**18.0**	4.0	0.220	1.0	99.0
**18.0**	**Stop Run**			
**18.0**	**Column Equilibration**			

10 min gradient

Time (min)	Duration (min)	Flow rate (μl/min)	% Solvent A	% Solvent B
**0.0**	0.0	0.220	99.0	1.0
**0.5**	0.5	0.220	99.0	1.0
**1.0**	0.5	0.220	92.0	8.0
**8.0**	7.0	0.220	80.0	20.0
**10.0**	2.0	0.220	65.0	35.0
**10.0**	**Column Wash**			
**14.0**	4.0	0.220	1.0	99.0
**14.0**	**Stop Run**			
**14.0**	**Column Equilibration**			

Supplemental Table S2. MS settings for DIA LC-MS/MS analysis

Settings	Full Scan	DIA (MS2)
**Resolution**	120000	60000
**AGC target (%)**	300	75
**Maximum injection time (s)**	Auto	118ms
**Scan range**	375-1200	See below
**HCD collision energy (%)**		30

Precursor mass range (m/z)	Isolation window (m/z)	Number of scan events
**375-500**	25	4
**500-600**	13	7
**600-800**	8	31
**850-900**	13	3
**900-1200**	25	9

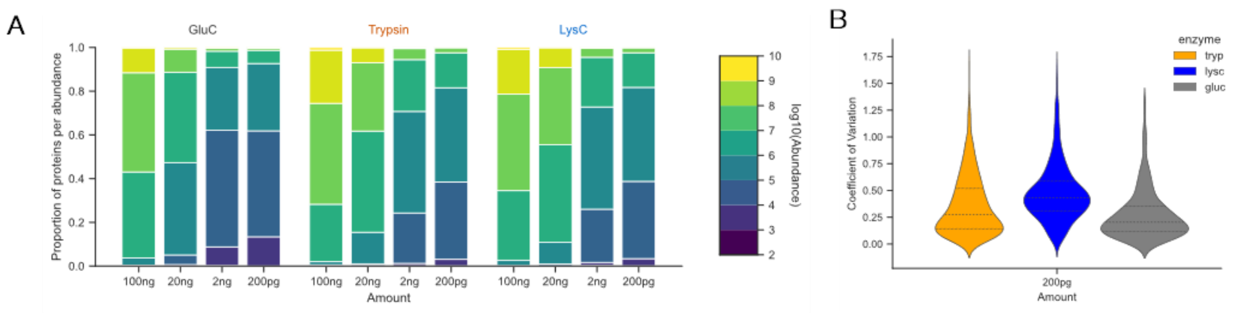

Supplemental Figure S1. Further characterization of proteomic data from different proteases (GluC, Trypsin, LysC). A. Proportion of proteins per abundance across dilution series. B. Plot of CV distributions for the three enzymes at a single cell load.

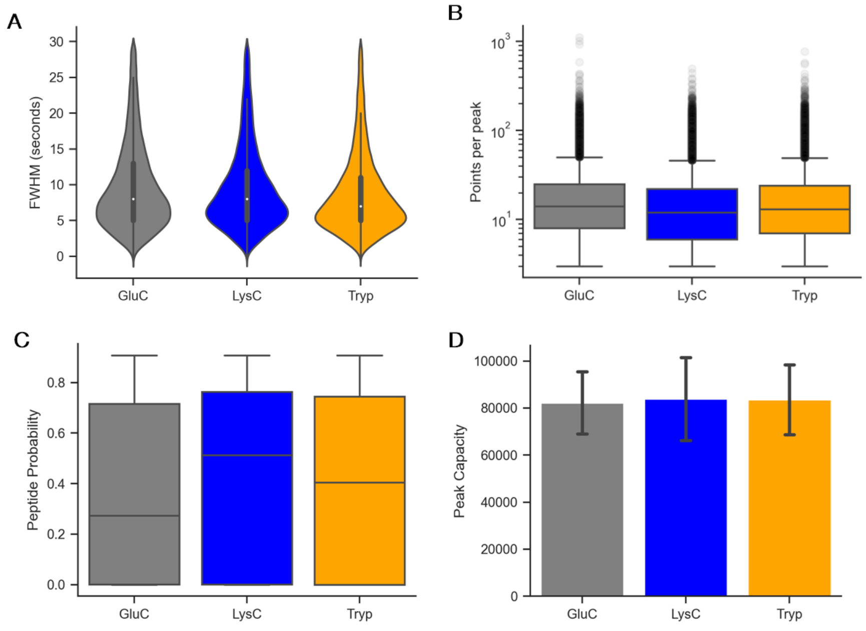

Supplemental Figure S2. LCMS experimental details from different proteases (GluC, LysC, trypsin). A. Full-width half maximum (FWHM) in seconds. B. Points per peak. C. Peptide Probability and D. Chromatographic peak capacity.

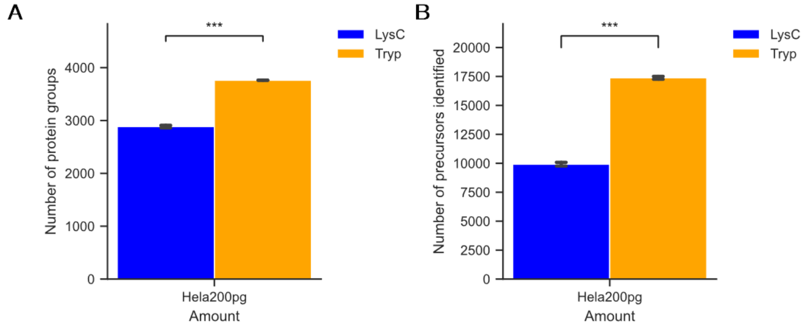

Supplemental Figure S3. DIA data from different proteases (LysC, trypsin). A. Number of protein groups. B. Number of peptide precursors identified.

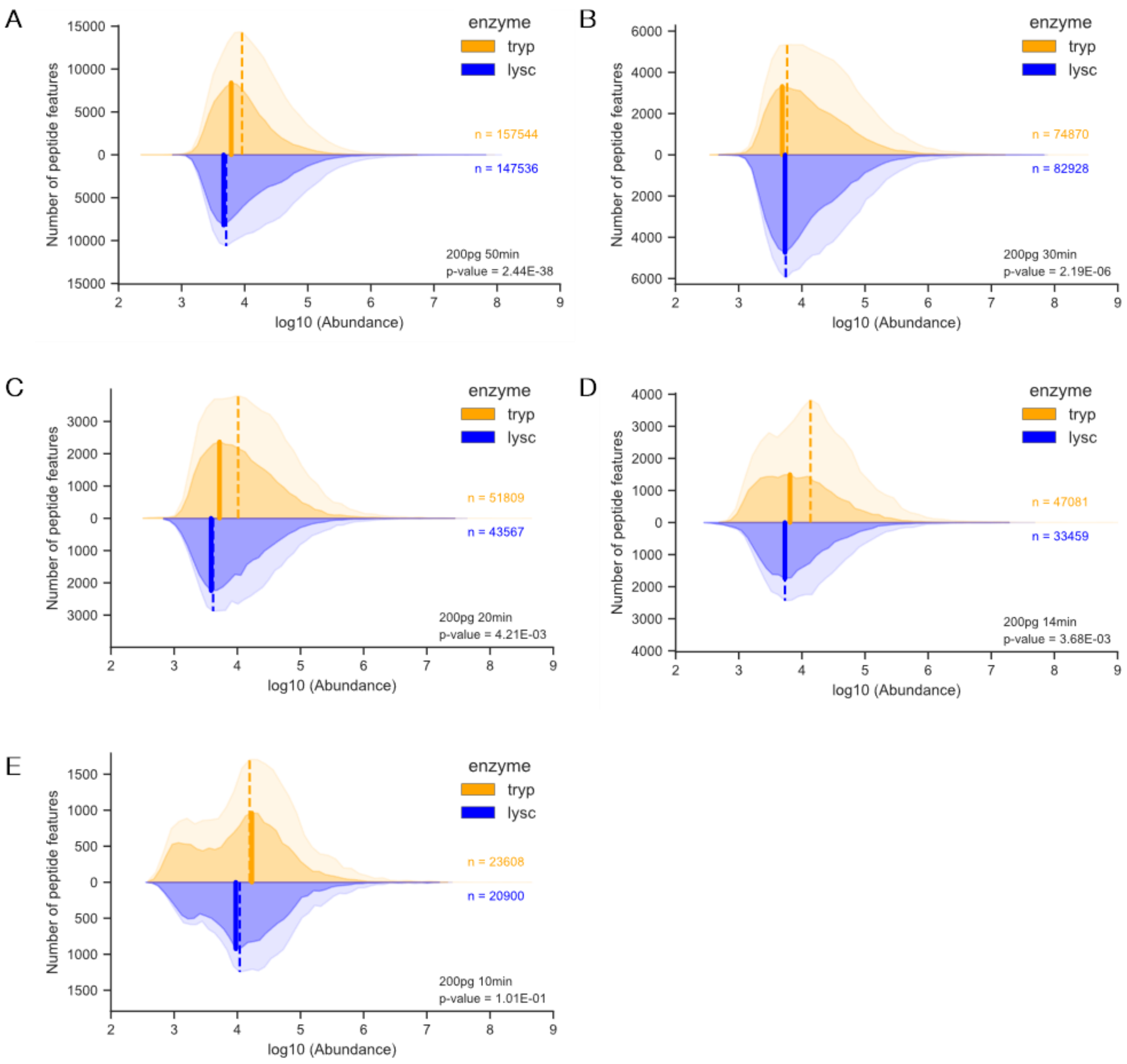

Supplemental Figure S4. Further characterization extraction and identification of peptide features across various acquisition times of A. 50 min, B. 30 min, C. 20 min, D. 14 min and E. 10 min.

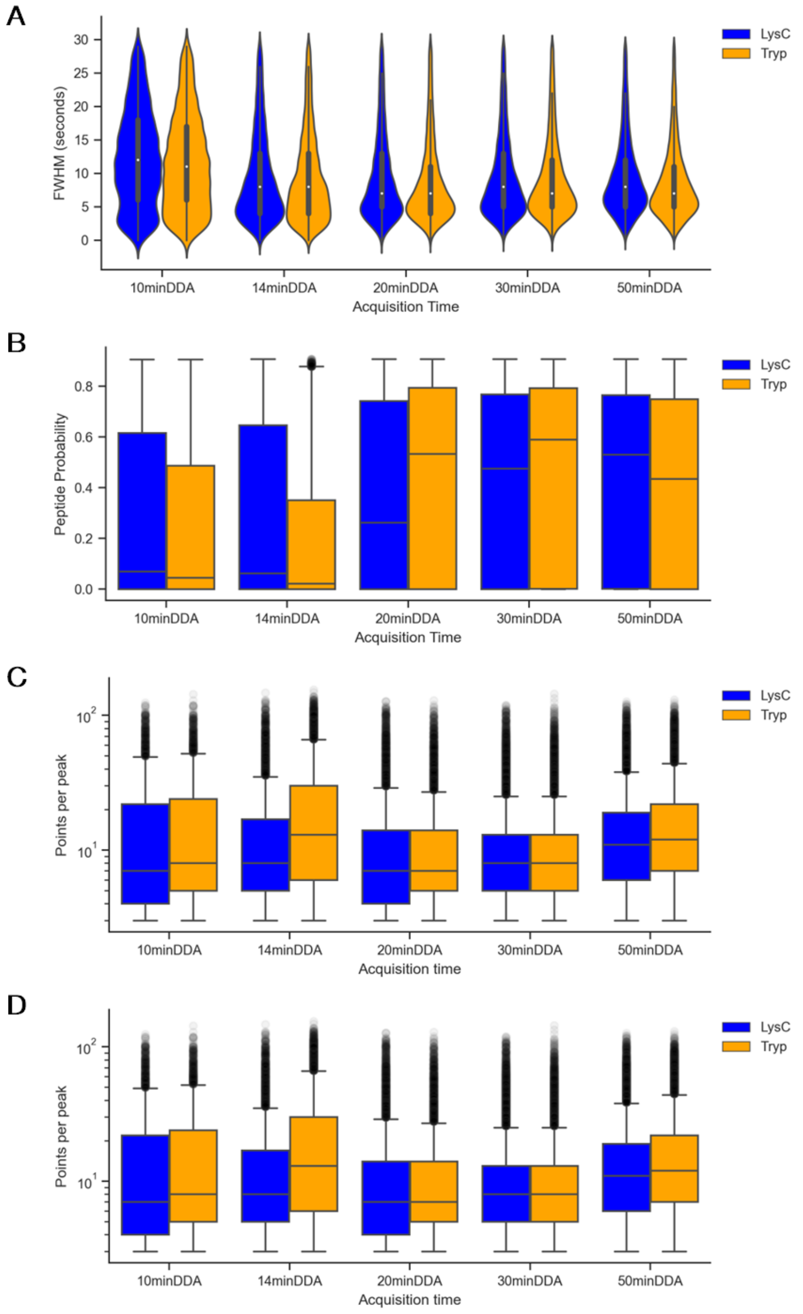

Supplemental Figure S5. LCMS experimental details from different proteases (LysC, trypsin) across various acquisition times of 10min, 14min, 20min, 30min and 50min. A. Full-width half maximum (FWHM) in seconds. B. Points per peak. C. Peptide Probability and D. Chromatographic peak capacity.

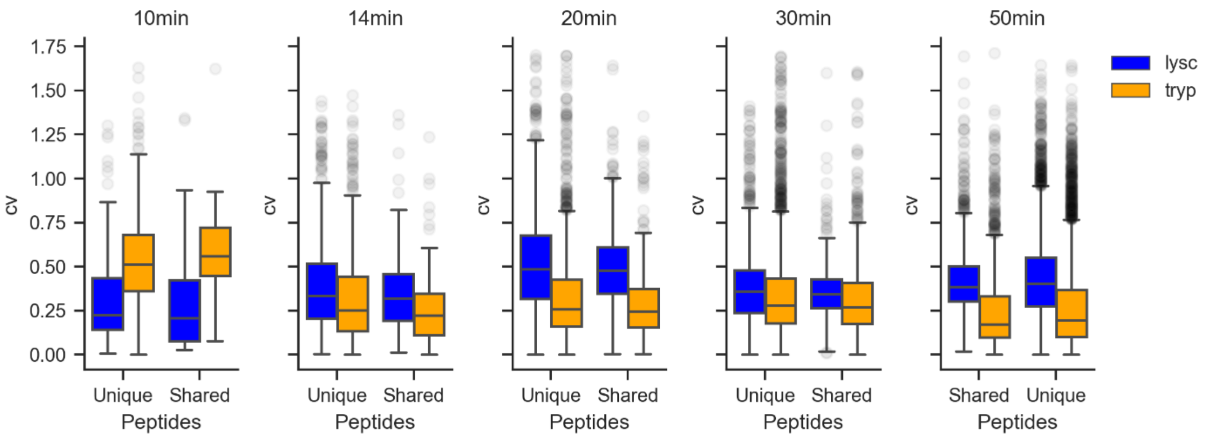

Supplemental Figure S6. CV distributions of shared and unique peptides across various acquisition times of 10 min, 14 min, 20 min, 30 min and 50 min.

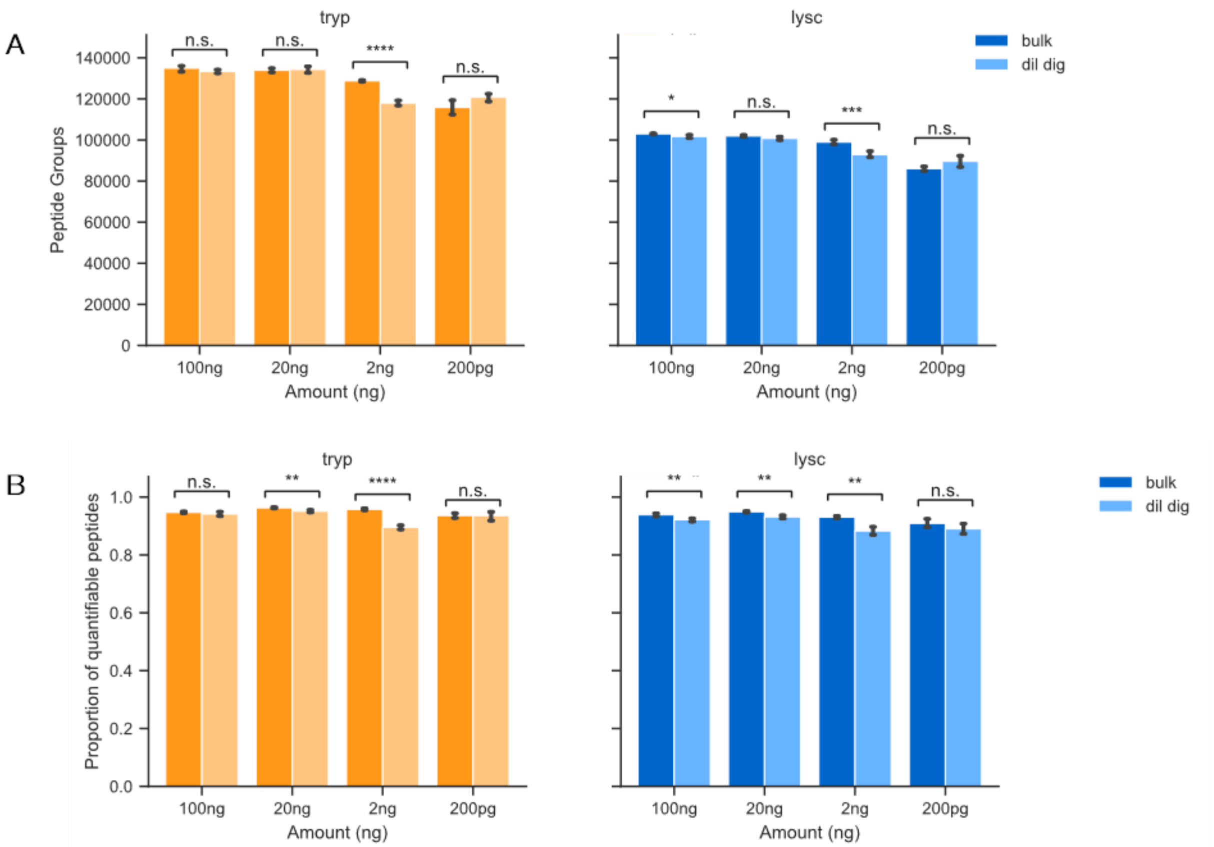

Supplemental Figure S7. Further characterization of proteomic data from Tryp and LysC, comparing the dilute-then-digest (lighter color) and bulk digest (darker color) methods. A. Number of peptide groups across dilution series. B. Proportion of quantifiable peptides across dilution series.

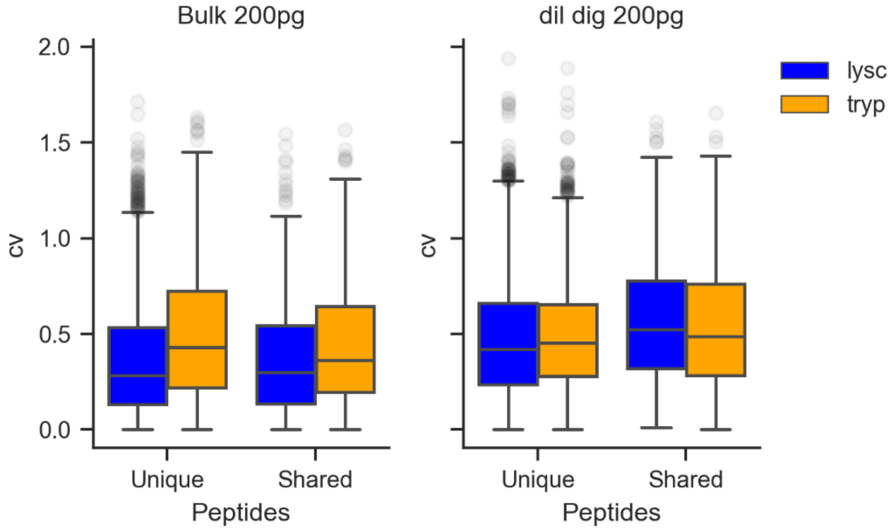

Supplemental Figure S8. CV distribution for peptides that shared or are unique between LysC and trypsin for bulk digest (left) and dilute-then-digest (right) methods at 200pg level.

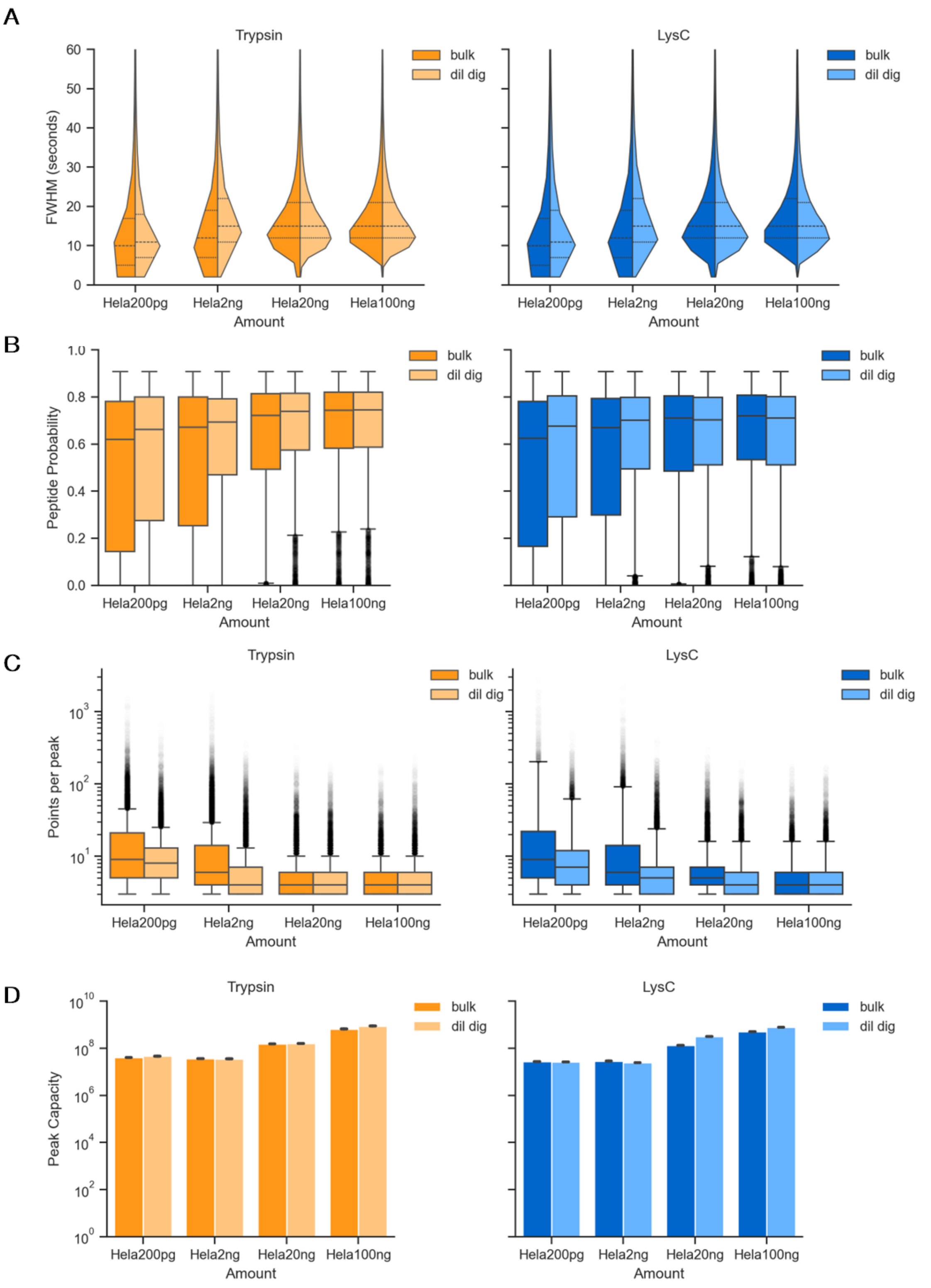

Supplemental Figure S9. LCMS experimental details from bulk digest and dilute-then-digest methods across various input loads at digestion. A. Full-width half maximum (FWHM) in seconds. B. Points per peak. C. Peptide Probability and D. Chromatographic peak capacity.
